# Late onset sciatalgia as a rare complication of percutaneous vertebroplasty; a case report

**DOI:** 10.4076/1757-1626-2-7960

**Published:** 2009-08-25

**Authors:** Farzad Omidi-Kashani, Mohamadhosein Ebrahimzadeh, Mohamadtaghi Peivandy

**Affiliations:** 1 Department of Orthopedic, Ghaem Hospital, Orthopedic Research Center, Mashhad University of Medical SciencesMashhadIran; 2 Department of Orthopedic, Emdadi Hospital, Mashhad University of Medical SciencesMashhadIran

## Abstract

Nowadays percutaneous vertebroplasty is commonly used for vertebral osteoporotic compression fractures. This technique is also useful for other lytic vertebral lesions including hemangioma. Although cement leakage is the most common technical complication, its clinical prevalence is rare and the presentation is always acute. Hereby, we reported a case of vertebral hamangioma in a 14-year-old girl treated by this technique and presented with late onset sciatalgia. According to our knowledge, such a late neurologic presentation has not been reported so far.

## Introduction

Vertebral hemangiomas are the most common benign vascular lesions of the spine. They are most common after the age of 40 but can be seen at any age. Male and female are affected equally. The site most commonly involves is thoracolumbar or lumbar area [1].

Most of these lesions are asymptomatic and diagnosed incidentally. On rare occasion a patient may present with back pain, deformity (kyphosis or scoliosis) following compression fracture, or neurologic symptoms [2].

## Case presentation

**Figure 1.**
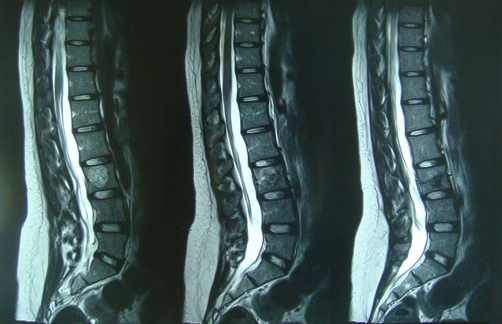
Preoperative imaging. MRI of the patient with L_3_ hemangioma. Note a silent fracture that was missed preoperatively.

A 14-year-old Asian girl residing on the north of Iran presented to our clinic with chronic low back pain from one year ago. She had a history of prolonged conservative treatment by home-made drugs and numerous bone setters. On plain radiograph, the only abnormal finding was a vertebral hamangioma of L_3_ that was confirmed by CT and MRI (Figure 1). The patient was offered a classic course of conservative treatment and physiotherapy for about 2 months. The Pain did not decrease and finally due to tumoral size (fear of impending fracture) and preference of the patient’s father, the bone augmentation surgery was suggested and carried out.

Immediately after percutaneous vertebroplasty (PVP) the pain was disappeared completely and the patient was discharged next day. After three days she returned to the school and did all her activities of daily living without any problems. This state lasted for 2 months. Then, gradually a radicular right leg pain was appeared and progressed unremittingly.

After 12 months of the procedure, the severity of the pain was so severe that the patient has given up going to the school. She waked up on the average of 3 to 4 times a night. The pain was radiated to the right ankle area with no associated low back pain. Physical examination of the patient revealed a positive SLR at 30°, weakness of the left ankle dorsiflexion (3/5) but any paraesthesia or deep tendon reflex change was not noted.

**Figure 2.**
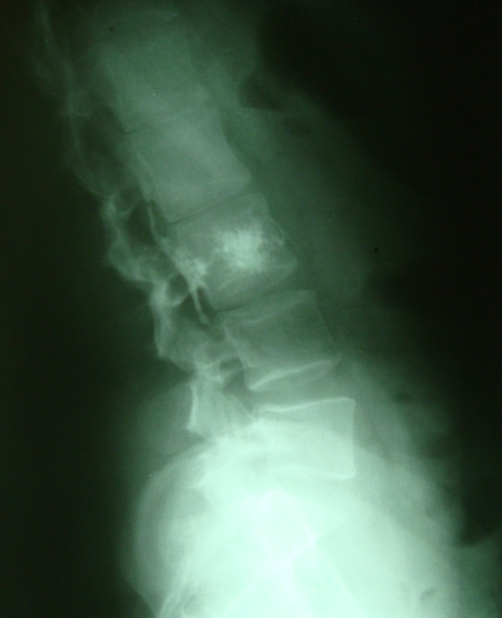
Plain radiograph after vertebroplasty. Posterior cement leakage is obvious.

**Figure 3.**
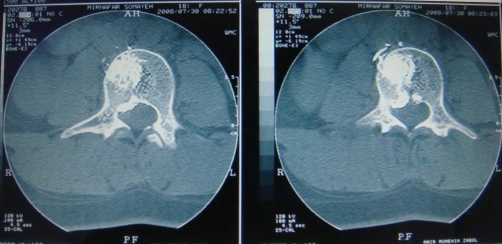
CT scan after vertebroplasty. Cement extrusion through the defect in posterior vertebral body is noted.

On postoperative plain radiograph and CT scanning, right-sided cement leakage and L_3-4_ neuroforamen involvement were noted (Figures 2 and 3). Electrodiagnostic study was compatible with chronic moderate right L_4_ and L_5_ demyelinating radiculopathy.

With retrospective analysis of the preoperative images (Figure 1), we got aware of a silent fracture through the vertebral body we injected cement. At first, we mistook it with a natural defect in the posterior vertebral cortex (blood vessels defect). Due to the severity and refractory nature of the symptoms, decompressive surgery was carried out with limited laminotomy and foraminotomy. The cement pieces extruded posterolaterally were excised completely and the L_4_ root was decompressed completely.

Immediately after surgery the pain was omitted but muscular power took about 1 month to return to the normal state. Now, the patient is in her 6^th^ month follow-up and symptom-free thoroughly.

## Discussion

Most of the vertebral hemangiomas are diagnosed during image evaluating of a patient for another concern. The typical appearance of these tumors on plain radiograph and CT scan are honeycomb (or celery stalk) and polka dot, respectively. These are due to prominent vertical striations of thickened trabeculae within vertebral body [3].

Most of the vertebral hemangiomas do not need any treatment but clinical observation. For symptomatic lesions, the traditional treatment of choice is radiotherapy due to radiosensitive nature of the tumor [4]. Nowadays, Open surgery is rarely indicated with these lesions except for some cases with spinal cord compression. The most significant concern about open surgery of these lesions is severe hemorrhage. Bleeding from these highly vascular tumors can be effectively minimized by preoperative angiography associated with selective embolization [5].

Intralesional ethanol injection has been reported in the treatment of vertebral hemangiomas and some authors claim of its effectiveness [6].

According to Hee HT, the classic indication for PVP is severe, continuous, and localized back pain relating to one or more collapsed vertebral bodies not responding to usual conservative therapy of 4 to 12 weeks’ period [7]. Therefore, the patient with vertebral fracture due to idiopathic osteoporosis, multiple myeloma, bone metastasis, hamangioma and other benign or malignant vertebral tumors may significantly improved clinically with this new and relatively safe technique [8].

The complications rate after PVP is very low but devastating. These include transient fever, mild intraoperative hypotension, temporary worsening of the fracture pain, infection, rib or adjacent segment fracture, cement leakage, neurologic deficit, and vascular involvement [9,10]. In fact, cement leakage is the most common technical complication but clinically asymptomatic in nearly all patients. According to our best knowledge, in those neurologically symptomatic cases, the presentation is always acute and from this point our case is unique. The reason for this late onset presentation is unknown for us, although. To prevent rare significant neurologic deficit associated with PVP, intact posterior vertebral body cortex is one of the most important prerequisite that must be thoroughly confirmed preoperatively.
